# Advancing Renal Amyloidosis Care: The Role of Modern Diagnostic Techniques with the Potential of Enhancing Patient Outcomes

**DOI:** 10.3390/ijms25115875

**Published:** 2024-05-28

**Authors:** Charlotte Delrue, Amélie Dendooven, Annelore Vandendriessche, Reinhart Speeckaert, Sander De Bruyne, Marijn M. Speeckaert

**Affiliations:** 1 Department of Nephrology, Ghent University Hospital, 9000 Ghent, Belgium; charlotte.delrue@uzgent.be; 2 Department of Pathology, Ghent University Hospital, 9000 Ghent, Belgium; amelie.dendooven@uzgent.be (A.D.); annelore.vandendriessche@uzgent.be (A.V.); 3 Faculty of Medicine, University of Antwerp, 2610 Wilrijk, Belgium; 4 Department of Dermatology, Ghent University Hospital, 9000 Ghent, Belgium; reinhart.speeckaert@uzgent.be; 5 Department of Laboratory Medicine, Ghent University Hospital, 9000 Ghent, Belgium; sander.debruyne@uzgent.be; 6 Research Foundation-Flanders (FWO), 1000 Brussels, Belgium

**Keywords:** amyloidosis, immunofluorescence, immunohistochemistry, mass spectrometry, proteomics, next-generation sequencing

## Abstract

Renal amyloidosis is a set of complex disorders characterized by the deposition of amyloid proteins in the kidneys, which causes gradual organ damage and potential kidney failure. Recent developments in diagnostic methods, particularly mass spectrometry and proteome profiling, have greatly improved the accuracy of amyloid typing, which is critical for disease management. These technologies provide extensive insights into the specific proteins involved, allowing for more targeted treatment approaches and better patient results. Despite these advances, problems remain, owing to the heterogeneous composition of amyloid proteins and the varying efficacy of treatments based on amyloid type. Access to sophisticated diagnostics and therapy varies greatly, highlighting the global difference in renal amyloidosis management. Future research is needed to investigate next-generation sequencing and gene-editing technologies, like clustered regularly interspaced short palindromic repeats (CRISPR), which promise more profound insights into the genetic basis of amyloidosis.

## 1. Characterizing Proteomic Variations in Amyloidosis

Amyloidosis is the accumulation of misfolded proteins outside cells in an insoluble β-pleated sheet form. When seen under polarized light and stained with Congo red, these amyloid deposits can be identified by their distinctive apple-green–orange birefringence. Electron microscopy has shown that these deposits comprise rigid, non-branching fibrils measuring 7.5 to 10 nm in diameter [1,2]. Localized amyloidosis arises when amyloids are produced and deposited in the same place. In systemic amyloidosis, amyloidogenic proteins are synthesized in one region, such as the bone marrow or liver, and then deposited in another, such as the heart or kidneys [3]. The mechanisms that cause amyloidosis, whether systemic or localized, are generally classified into three broad categories: (1) the increased production of proteins that result in a predisposition toward amyloid formation, (2) mutations in proteins that increase the likelihood of misfolding compared to their normal counterparts, and (3) a natural proclivity of certain normal (wild-type) proteins to form amyloid [4]. Systemic amyloidosis can be hereditary or acquired. Notably, the absence of a family history does not preclude hereditary amyloidosis. Approximately half of the people with hereditary amyloidosis have no known family history of the disease [4,5,6].

**Figure 1:**
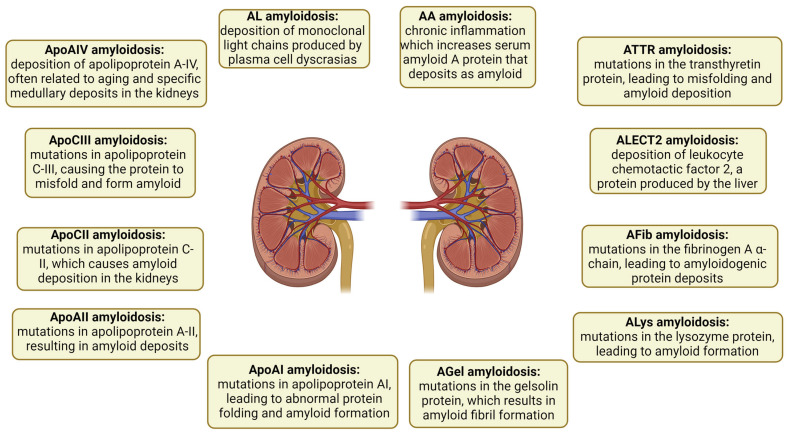
Subtypes of renal amyloidosis and their pathogenic mechanisms. Each subtype is depicted with its respective pathogenic mechanism, highlighting the diversity of protein deposits involved in renal amyloidosis. AA: serum amyloid A; AL: immunoglobulin light chain amyloidosis; ApoAI: apolipoprotein AI; ApoAII: apolipoprotein A-II; ApoAIV: apolipoprotein A-IV; ApoCII: apolipoprotein C-II; ApoCIII: apolipoprotein C-III; ATTR: transthyretin; Fib: fibrinogen; Gel: gelsolin; LECT2: leukocyte chemotactic factor 2; Lys: lysozyme.

Amyloidosis is classified based on the originating protein and is typically categorized as either a wild-type (acquired) or germline pathogenic variant (mutant). Proper amyloid typing is vital to avoid diagnostic mistakes [4,5,6]. Forty-two proteins are amyloidogenic, with at least 17 being capable of causing systemic disease [7]. Presently, six amyloidogenic proteins have the ability to form amyloids in both the wild-type and mutant forms. These proteins include transthyretin (ATTR), beta-2 microglobulin (Aβ2M), serum amyloid A (AA), apolipoprotein A-IV (ApoAIV), Aβ protein (Aβ), and prion protein (APrP) [8,9]. Other examples of proteins include leukocyte chemotactic factor 2 (ALECT2), fibrinogen α-chain (AFib), gelsolin (AGel), apolipoproteins A-I and A-II (AApoAI and AApoAII), and apolipoprotein C-III (AApoCIII) [10]. The subtypes of amyloidosis and their respective pathogenic mechanisms are illustrated in Figure 1.

Besides amyloidogenic proteins, multiple other proteins have been identified that contribute to our understanding of the physiology of the disease. Proteins such as clusterin, vitronectin, apolipoprotein E (ApoE), ApoA4, and serum amyloid P-component (SAP) constitute amyloid signature proteins found in all types of amyloidosis and across all affected tissues. While some proteins, such as SAP, may promote amyloid development, others, such as clusterin, may function as amyloid chaperones and reduce amyloid production. Amyloid plaques contain proteins from the affected organs, which can provide information about the degree of amyloid accumulation, the processes that lead to cell damage, and the physiological reactions of various tissues to amyloid accumulation [11]. Proteins deposited in amyloid plaques (expanded proteome) and proteins overexpressed in plaques compared to controls (plaque-specific proteome) have been investigated in 2650 cases from eight different types of kidney amyloidosis [immunoglobulin light chain (AL), heavy chain (AH), a mix of heavy and light chain amyloid (IGH), ALECT2, AA, AFib, AApoAIV, and AApoCII] using laser microdissection–mass spectrometry. The proteomic content was higher in the more indolent types of amyloid (non-AL) than in AL [12]. The proteomic “density” of amyloid plaques can serve as an indicator of more gradual disease progression [13]. AA, AFib, and AApoCII amyloidosis were the most proteomically distinct, driven by increased complement pathway proteins, whereas AL and ALECT2 showed significant proteomic heterogeneity [14]. 

Complement pathway proteins are important sources of diversity across various amyloidosis types and proteomic subgroups. They may be synthesized in situ and may signify an unusual response to pathogens. A noticeable reduction in ubiquitins and heat shock proteins in patients with AL, AH, AApoCII, and AFib was noted, which may reflect the overutilization of these proteins by the ubiquitin–proteasome system [14]. This may also apply to heat shock proteins, a widely recognized cluster of chaperones that counteract amyloid formation [15]. Ubiquitins and heat shock proteins can be directly captured within aggregates of misfolded proteins, further exacerbating the collapse of cellular proteostasis [16,17,18]. 

The proteome specific to kidney AL plaques comprised 24 proteins, including those linked to kidney damage (α1 antitrypsin and heat shock protein β1). Hierarchical clustering of AL cases using their plaque-specific proteomes revealed four distinct clusters. One cluster, associated with better kidney survival, was distinguished by its higher proteomic content and the presence of 14-3-3 proteins; yet, it had reduced levels of amyloidogenic light chains (LCs) and the majority of signature proteins. The 14-3-3 proteins are a family of structurally similar phospho-binding proteins that regulate major cellular functions and act as molecular chaperones for several other proteins involved in a variety of cellular processes such as signal transduction, proliferation, differentiation, apoptosis, and autophagy [19]. They interact directly with different forms of amyloid and help to eliminate them [20,21]. Proteins linked to kidney diseases, including heat shock protein family B (small) member 1 (HSPB1), S100A6, transgelin (TAGLN), and TIMP metallopeptidase inhibitor 3 (TIMP3), were shown to be enriched in the proteome unique to AL. HSPB1 enhances autophagy during acute kidney injury (AKI) and is similarly activated by misfolded proteins [22]. S100A6, a protein that binds calcium, is elevated in AKI and potentially reduces β-amyloid accumulation in animal studies of Alzheimer’s disease [23,24]. TAGLN associates with cytoskeletal proteins and is upregulated by the amyloid precursor protein in Alzheimer’s disease [25]. Lastly, TIMP3, a component also found in cardiac amyloid proteome, may play a role in matrix remodeling and fibrosis in AL [13]. Glutathione-S transferase (GST) and phosphatidylethanolamine-binding protein 1 (PEB1) showed the highest upregulation in the proteome of early-stage amyloidosis. Both function as antioxidants, and PEB1 plays a key role in regulating the ferroptosis pathway [26,27]. Finally, the accumulation behavior of amyloidogenic proteins is independent of other proteins, suggesting that kidney toxicity in this condition cannot be solely attributed to amyloid levels. Investigating the functions of the proteins regarding disease development could provide a foundation for creating new treatments [12].

## 2. Diagnostic Techniques

### 2.1. Current Challenges in Diagnosis and Treatment

Diagnosing systemic amyloidosis involves identifying symptoms indicative of amyloidosis, along with tissue confirmation. Fat aspiration is commonly used for diagnostic purposes because of its accessibility. However, the success rate of this method varies by amyloid type, with detection rates ranging from 15% to 80%. The effectiveness of fat aspiration also relies on the expertise of the pathologist and the quantity of fat collected [28,29]. Other common sources for tissue sampling include bone marrow biopsy (conducted if a monoclonal protein is detected), minor salivary glands, rectal biopsy, and biopsies of any organs that are affected [10]. 

Renal amyloidosis is difficult to diagnose and treat because of the variable composition of amyloid proteins and variety of clinical symptoms. The multiplicity of amyloidosis forms makes diagnosis difficult because each requires a unique treatment and has a different prognosis. The complexities of the initial symptoms of renal amyloidosis, such as proteinuria and renal insufficiency, are significant in its diagnosis. This can cause delays in identification until significant kidney damage occurs. Furthermore, established diagnostic techniques like Congo red stains and IHC have inadequate sensitivity and specificity. The latter may fail to distinguish between amyloid fibril types, which is critical for effective therapy planning. IHC is commonly used in pathology laboratories to identify the natural forms of proteins. This focus may limit the sensitivity and specificity of the technique for detecting misfolded proteins such as amyloid fibrils. This specificity issue arises because IHC antibodies are frequently intended to recognize normal protein conformations, thereby missing or underdetecting the altered structures observed in disease conditions. Recent developments, such as MS, offer greater precision, but are not commonly available and need specialist equipment and knowledge, limiting their use exclusively to specialized centers [30]. While this limits their immediate accessibility, it is feasible to send biopsies to specialized laboratories for analysis. It is impractical for all laboratories within a country to provide the full spectrum of specialized methods needed for comprehensive coverage. Therefore, establishing one specialized laboratory per country, or in some cases just a few laboratories for the entire region of Europe, could suffice for advanced diagnostics such as next-generation sequencing (NGS) or MS testing. This would ensure that all patients have access to the necessary diagnostic methods regardless of their location, thereby improving the overall diagnosis and treatment of amyloidosis.

Treatment of renal amyloidosis is very difficult, owing to the lack of a cure and the fact that most medications focus on symptom management and halting progression. The efficacy of treatment varies greatly depending on the type of amyloidosis. For example, patients with AL amyloidosis may benefit from chemotherapy or stem cell transplantation to reduce amyloidogenic light chain synthesis. In contrast, patients with ATTR amyloidosis may be given Tafamidis, which stabilizes the transthyretin tetramer and prevents it from dissociating and misfolding. However, the efficiency of these treatments varies and often comes with significant adverse effects. Furthermore, there are no particular treatments for other rarer kinds of amyloidosis, such as ALECT2, which affects only a few ethnic groups and has no targeted therapeutics [31]. 

The global difference in access to sophisticated diagnostics and therapies is a substantial hurdle, particularly in underserved areas where the disease may be underdiagnosed or misdiagnosed, and adequate care options are limited. This necessitates international collaboration to improve diagnostics and treatment accessibility, assuring equitable health outcomes for people with renal amyloidosis worldwide.

### 2.2. Recent Advancements in Diagnostic Techniques

Amyloid deposits are histologically similar across all types of amyloidosis, making amyloid typing a critical aspect of the diagnostic process with significant diagnostic, therapeutic, and prognostic implications. The first approach in typing renal amyloidosis is direct immunofluorescence (IF) on frozen tissue (Table 1). For example, AL amyloidosis is characterized by strong staining for a single immunoglobulin light chain (LC) but no staining for heavy chains (HCs) [32,33]. IF has limits in terms of sensitivity and specificity, notably in the diagnosis of immunoglobulin heavy and light chain (AHL) and immunoglobulin heavy chain (AH) amyloidosis [32,34]. 

Immunohistochemistry (IHC) can be used with immunoperoxidase and antibodies against known amyloid precursor proteins such as SAA, ALECT2, and ATTR [37]. This method might cause extensive background staining, making it less effective than IF in certain situations. Both IF and IHC can result in false positives and negatives [37,38]. For example, in AL amyloidosis, renal amyloid deposits may be negative for both κ and λ LCs in a significant number of cases [32,35,36]. 

The introduction of new diagnostic tools, particularly mass spectrometry (MS) proteomic-based analysis, has dramatically altered the landscape of renal amyloidosis diagnosis. MS provides great precision and can reliably identify the kind of amyloid protein present, which is critical for developing effective treatment options [30]. It directly identifies the specific protein subunit within the deposit along with associated universal amyloid proteins such as apolipoprotein E, serum amyloid P, and apolipoprotein A-IV. Amyloid typing primarily requires the combination of laser capture microdissection and MS, while the characterization of amyloid only requires MS. MS offers nearly 100% sensitivity and specificity, establishing it as a benchmark for amyloid typing. This approach is better than the conventional methods by identifying low concentrations of amyloid deposits and distinguishing between similar types of amyloid proteins that appear identical under microscopy but have different clinical implications [42]. However, MS is costly, necessitates specialist equipment, and is not readily available. Therefore, antigen-antibody-based procedures such as IHC, IF, and immunoelectron microscopy, while less accurate and sensitive than MS, are viable alternatives, especially when performed in trained laboratories. MS is commonly employed in situations where other methods fail or produce equivocal results [28,39,40]. Recent improvements have also introduced proteome profiling, which not only improves diagnostic precision, but also helps to understand the etiology of various amyloidosis subtypes [40]. 

NGS is another state-of-the-art method in the diagnosis of inherited forms of amyloidosis. Large DNA segments or entire genomes can be sequenced using NGS to identify genetic variations that may predispose individuals to specific types of amyloidosis. This genetic information is very helpful for familial cases, where early diagnosis may lead to preventive therapy approaches [5,41].

## 3. Diverse Forms of Amyloidosis: Classification and Characteristics

### 3.1. AL Amyloidosis

A plasma cell abnormality that results in an excessive generation of defective immunoglobulins is the etiology of AL amyloidosis (Table 2) [43]. These faulty proteins combine to produce AL amyloids, which are β-pleated, insoluble fibrils that accumulate in different organs [44,45]. 

The first step in identifying AL amyloidosis is the detection of circulating monoclonal LCs. Serum-free light chain (FLC) assays, the quantification of light chains (or free light chains) in urine, 24 h urine protein electrophoresis with immunofixation, and serum protein electrophoresis with immunofixation can be used to identify amyloidogenic monoclonal components (Table 3). The concentrations of light chains in urine from amyloidosis patients may be very low. Laboratories need to review the gels very carefully and use high-resolution systems. Just running urine electrophoresis and immunofixation is not sufficient, and sensitive methods must be employed by the lab. The chance of diagnosing AL amyloidosis is considerably decreased if a monoclonal component is not discovered [65]. 

Kidney and cardiac impairment are the most frequent consequences in people with AL amyloidosis. However, it must be emphasized that the heart is the most frequently involved organ in most series, provided its key prognostic impact [44,45]. About 25% of cases advance to end-stage kidney disease (ESKD), requiring renal replacement therapy including dialysis or kidney transplantation. Approximately two-thirds of cases have kidney involvement, which frequently presents as nephrotic syndrome [1,71]. Moreover, a variety of nonspecific symptoms, such as fatigue, inadvertent weight loss, arrhythmias, numbness, paresthesia, pain, and macroglossia are frequently observed in patients with AL amyloidosis. The average duration from symptom onset to diagnosis for AL amyloidosis is two to four years [72]. These broad and varied symptoms sometimes cause delays in diagnosis. Patients who experience this delay in diagnosis frequently present with irreversible organ damage, such as progressive heart failure, ESKD, and potentially fatal consequences [73]. 

Although kidney involvement may not affect overall survival as significantly as cardiac involvement, existing renal staging models effectively predict the risk of patients progressing to ESKD. The Palladini criteria primarily uses two parameters: proteinuria and eGFR. Patients are divided into stages based on the probability that they may develop ESKD. The accuracy with which it forecasts renal outcomes demonstrates its importance in managing AL amyloidosis involving the kidney. The Pavia approach indicates that an eGFR of <50 mL/min and proteinuria of >5 g/24 h are high-risk indicators for the progression of dialysis. Patients with proteinuria below this cutoff and higher eGFR are classified as low-risk and have reduced rates of ESKD development. This system also introduced the criteria for renal response and progression, which are critical for evaluating treatment efficacy. A good renal response, which is associated with better kidney survival, is defined as a reduction in proteinuria of at least 30% or <0.5 g/24 h without the progression of kidney insufficiency. To further assist with patient management and trial design, a criterion for renal advancement is the adoption of a ≥25% decline in eGFR within a six-month period [74]. The Kastritis criteria also focus on the renal outcomes of patients with AL amyloidosis. Three groups were created based on the 24 h proteinuria-to-eGFR ratio (UPr/eGFR). Stage 1 was defined by a UPr/eGFR ratio of <30, stage 2 by a ratio of 30 to 99, and stage 3 by a ratio of ≥100. Over a three-year period, the rates of progression to dialysis for patients in stages 1, 2, and 3 were 0%, 11%, and 46%, respectively. The prediction accuracy of the model for kidney survival has been validated, highlighting its importance in both research and clinical practice for patients with kidney-affected AL amyloidosis [75]. As suggested by Basset et al., the urine albumin/creatinine ratio (UACR) is another method to simplify the assessment of kidney involvement. Their model assessed UACR to diagnose, stage, and monitor renal AL amyloidosis, providing a practical alternative to the 24 h proteinuria collection. The utility of UACR in predicting dialysis need and kidney survival makes it another option for clinicians managing AL amyloidosis [76]. 

### 3.2. Serum Amyloid a Amyloidosis (AA)

AA amyloidosis is associated with chronic inflammatory conditions, infections, and familial diseases such as Familial Mediterranean Fever (FMF) [77]. It results from the prolonged elevation of serum amyloid A protein, an acute-phase reactant synthesized by the liver during inflammation [55]. Proteinuria is the typical initial symptom of AA amyloidosis and can develop into nephrotic syndrome and chronic kidney disease (CKD). Although the disease mostly affects the kidneys, it can also affect the spleen and the liver. Kidney biopsy results revealed amyloid plaques that stained positively with Congo red and displayed apple-green birefringence under polarized light, confirming the diagnosis. IHC and MS have been used to ascertain whether the SAA protein was present in the deposits. The goal of treatment is to control underlying inflammatory conditions to reduce the generation of SAA [77,78].

Depending on the related illnesses, biologics, disease-modifying antirheumatic drugs (DMARDs), or anti-inflammatory medications may be used. In cases of ESKD, kidney transplantation may be explored, although there is a chance that the transplanted kidney will develop amyloidosis again. The management of the underlying inflammatory diseases has a major impact on the prognosis of AA amyloidosis. Amyloid deposits can be decreased and kidney function can be stabilized or improved with effective therapy of inflammatory diseases [77,78].

### 3.3. Transthyretin Amyloidosis (ATTR)

TTR is a tetrameric protein primarily synthesized in the liver, choroid plexus, and retinal pigment epithelium [79]. Amyloid fibrils are derived from the monomeric form of the protein, which can be wild-type (ATTRwt) or variant (ATTRv) owing to genetic mutations [80]. More than 120 amyloidogenic mutations have been identified in TTR. Common variants include V30M, which is prevalent in populations in Portugal and Japan; V122I, found predominantly in African Americans; and T60A in individuals of Irish descent. These mutations affect the clinical presentation and distribution of amyloid deposits in the body. ATTR amyloidosis commonly affects the heart and the nerves. Cardiac involvement is particularly noted in ATTRwt, which predominantly affects older men. Neuropathic symptoms, both autonomic and peripheral, occur in approximately 50% of cases [48]. Initially, kidney involvement was underrecognized, but recent studies have shown that a significant number of patients develop proteinuria and ESKD, especially those with specific pathogenic variants, such as V30M [46,47]. One study indicated that one-third of the patients with ATTR amyloidosis eventually developed proteinuria, with 10% progressing to ESKD [46]. Notably, women with the V30M mutation are more likely to develop nephrotic-range proteinuria and ESKD than men. CKD rates have increased from 16.5% to 30.4% over a median follow-up of 7.9 years, with late-onset symptoms and specific mutations such as V122I being associated with CKD [81]. 

Antisense oligonucleotides (ASOs) like Inotersen and Patisiran, which target the mutant TTR mRNA to limit its production, are one treatment strategy for ATTRv [82,83]. ATTRwt is treated with tacramidis, a TTR tetramer stabilizer [84]. Although their effects on kidney function have not been well-studied, both treatments try to manage symptoms and reduce the progression of the disease. New therapies like clustered regularly interspaced short palindromic repeats (*CRISPR*) gene editing have demonstrated potential in lowering serum TTR levels, thereby providing a long-term remedy for ATTRv management [85,86]. The effectiveness and safety of these innovative strategies need to be confirmed through clinical trials [7].

### 3.4. Leukocyte Cell-Derived Chemotaxin-2 Amyloidosis (ALECT2)

ALECT2 amyloidosis, characterized by deposits primarily in the kidneys and liver, has a notable ethnic predisposition and is more common in Hispanic, Middle Eastern, and South Asian populations [49,50,51,52]. Its pathogenesis involves the deposition of LECT2, a protein produced by the liver, although the precise pathological mechanisms remain unclear [32]. Subnephrotic-range proteinuria and varied degrees of kidney impairment are common presentations for patients. Although the disease usually progresses slowly, about one-third of individuals may end up with ESKD [87,88]. Due to the gradual course and vague symptoms of the disease, the diagnosis is frequently made too late, resulting in severe kidney impairment at the time of diagnosis. The main basis for the diagnosis is the results of the kidney biopsy, where the deposits of ALECT2 show up as amorphous eosinophilic material on Congo red staining and exhibit apple-green birefringence in polarized light. IHC verifies that LECT2 is present in the deposits. For a conclusive diagnosis, sophisticated methods like MS are essential, especially when differentiating ALECT2 from other forms of amyloidosis. For ALECT2 amyloidosis, no specific treatment is available. The main goals of care are symptom control and a decrease in the course of kidney disease. In several cases, kidney transplantation has been performed, with generally positive results and no documented recurrence of amyloid in the transplanted organ. According to another study, patients with ALECT2 amyloidosis had different prognoses. Some patients may experience years of stability, but others may develop ESKD, necessitating dialysis or kidney transplantation. The degree of organ involvement and the presence of concomitant diseases can affect the overall progression of the disease [3,7,52,89].

### 3.5. Fibrinogen a α-Chain Amyloidosis (AFib)

AFib amyloidosis is associated with pathogenic variants in the *Aα* gene of the fibrinogen protein, which has three subunits: α, β, and γ. Among the 18 pathogenic variants described, 15 are known to result in amyloid production [56,57]. These variations, which frequently result in a more aggressive disease course with early symptom onset and rapid development to ESKD, include frameshift mutations and substitutions of single and double bases [57]. Individuals who carry these genetic variants usually exhibit increasing CKD and nephrotic-range proteinuria. Once diagnosed, the median time from diagnosis to dialysis initiation is approximately 8 months. Renal pathology in AFib shows massive glomerular obliteration by amyloid material, generally sparing blood vessels [56]. Apart from kidney involvement, amyloid deposits have occasionally been documented in the heart, although echocardiographic or scintigraphy evidence of amyloid heart disease is unusual. Patients may also experience ischemic cardiac and cerebral events. Peripheral neuropathy is rare in these patients. No specific treatment is currently available for AFib. This condition often recurs following kidney transplantation [56,90,91]. However, recurrence has not been documented in patients who receive combined liver and kidney transplantation, where the morbidity and mortality rates are notably higher, thus necessitating careful patient selection. One reported case involved a pre-emptive liver transplant that successfully prevented the development of ESKD [92].

### 3.6. Lysozyme Amyloidosis (ALys)

Lysozyme, a bacteriolytic enzyme produced by macrophages, gastrointestinal tract cells, and hepatocytes, has over 10 identified pathogenic variants, all located in the β domain of the protein [67]. These mutations affect protein folding and stability and lead to amyloid formation. Originally believed to exclude nerve involvement, recent cases have documented peripheral neuropathy in individuals with specific variants, such as D87G and L102S, which also presented with cardiac, renal, and gastrointestinal symptoms [34,67]. ALys has several unique clinical features. Unlike other hereditary amyloidosis forms, kidney symptoms can appear early, even in adolescents, with ESKD occurring as early as the third decade of life. Symptoms typical of Sicca syndrome, such as dry mouth and dry eyes, occur early in the course of the disease. Common symptoms include diarrhea, abdominal pain, and poor appetite [67,68,90]. Severe complications involving bleeding, particularly in the gastrointestinal tract, are often associated with hepatic rupture in patients with the D67H variant and mesenteric lymph node hemorrhage in patients with the W64R variant [68,69,93]. Although less common, cardiac complications have been noted in a few cases, adding to the systemic impact of the disease [34,68]. No specific protein-directed therapy is currently available for patients with ALys. Management is primarily supportive, focusing on symptom control and the management of organ involvement [67,70]. Liver transplantation has been performed in cases of hepatic rupture, and, although amyloid recurrence has been detected via scintigraphy, it has not led to graft failure. Kidney transplantation outcomes have generally been positive, with no documented recurrence of amyloidosis, although follow-up periods have been short [70].

### 3.7. Gelsolin Amyloidosis (AGel)

Gelsolin is a calcium-sensitive protein that is pivotal for regulating the gelsolin transition of actin in the cytoplasm, and is essential for macrophage locomotion [94]. It exists in both cytosolic and secreted forms, and plays a role in modulating the inflammatory response. The genetic origins of AGel were first highlighted in 1969 when it was discovered in a Finnish family [95]. Two pathogenic variants have been detected to date: 654G>A, which is primarily observed in Denmark, the former Czechoslovakia, France, and Brazil; and 654G>T, which is primarily found in various populations such as the African American, Hispanic, European, Asian, and Finnish populations [96]. AGel typically begins in the third decade of life with symptoms such as corneal lattice dystrophy due to amyloid deposits [58]. Other common manifestations include cutis laxa and body hair loss. The disease progresses to affect multiple cranial nerves, leading to facial paralysis, sensory loss, tongue atrophy, and drooling due to the involvement of the facial, trigeminal, glossopharyngeal, and hypoglossal nerves. The peripheral and autonomic nerves are also affected. Clinical symptoms tend to be present earlier in women, who are more likely to exhibit ophthalmological symptoms than cutaneous and neurological symptoms, which are more common in men [59]. Kidney impairment and severe proteinuria are hallmarks of renal involvement, which typically happens later in life. A small percentage of patients in documented cases developed ESKD, and some of them needed transplants [94]. In certain instances, there has also been evidence of cardiac involvement. Remarkably, a thorough investigation involving 272 individuals with AGel revealed that, while kidney problems were more frequent and lethal malignancies were less frequent, overall life expectancy did not differ appreciably from that of the Finnish community [96]. There is no direct therapy available to treat AGel at the moment. The goal of management is to prevent problems from organ involvement and to relieve specific symptoms in a symptomatic and supportive manner [7].

### 3.8. Apolipoprotein A-I Amyloidosis (AApoAI)

ApoAI is a major component of high-density lipoproteins (HDLs) and is synthesized in the liver and intestine. This disease was originally identified in the Iowa family of British descent. To date, more than 20 pathogenic variants of ApoAI have been identified, each associated with different patterns of organ involvement and disease progression [53]. In a comprehensive UK study, the kidney was the most commonly affected organ (81% of cases), followed by the liver (67%) and the heart (28%) [54]. Other affected organs included the peripheral nerves (11%), larynx (11%), and testes (4%). The extent of heart involvement varies significantly, depending on the specific pathogenic variant. For instance, individuals with the pArg172Pro variant exhibited 100% cardiac involvement but only 16.7% had kidney involvement. Notably, a unique medullary deposition pattern of ApoAI amyloid, predominantly affecting the renal medulla with minimal glomerular deposits, has been identified in Italian patients with the Leu75Pro variant [97]. These patients typically present with declining kidney function in their 50s and little or no proteinuria. Kidney and other organ transplants have been performed in patients with AApoAI, with documented cases of amyloid recurrence in the heart and kidney allografts. However, the transplanted organs remained functional [98]. It is interesting to note that two relatives who received kidney transplantation alone also had retinal involvement, which is defined by the atrophy of the retinal pigment epithelium as a result of choriocapillaris obliteration [99]. It is now understood that this retinal alteration could be an organ of interest for AApoAI. Individuals who received a combined liver and kidney transplant did not develop any recurrences, and scintigraphy even revealed a decrease in amyloid load. More research is being carried out on the possibility of using liver transplantation to stop the deterioration of other organs caused by AApoAI. In afflicted patients, this strategy might provide a means of lowering the systemic amyloid burden [100].

### 3.9. Apolipoprotein A-II Amyloidosis (AApoAII)

Apolipoprotein AII (ApoAII) is a major component of high-density lipoproteins, synthesized in the liver and intestine [101]. This form of amyloidosis was first identified in 2001 and has been associated with several pathogenic variants. Although most pathogenic variants are caused by a nonsense mutation, non-stop codon variants have also been documented. The progression of kidney dysfunction and proteinuria are common presentations in AApoA II patients. Nevertheless, amyloid deposits have been discovered in a number of organs, including the skin, rectum, adrenal glands, liver, spleen, bone marrow, and heart. AApoAII is histologically distinguished from AApoAI by substantial deposition in the glomeruli. Patients with AApoAII have a documented risk of amyloidosis recurrence in their transplanted kidneys, which is an important factor in the treatment and prognosis of this disease [60].

### 3.10. Apolipoprotein C-II (AApoCII) Amyloidosis

Important forms of ApoCII include chylomicrons, very-low-density lipoprotein (VLDL), and HDL particles [63]. It is essential for the metabolism of lipids because it serves as a cofactor for lipoprotein lipase, an enzyme that breaks down triglycerides. Patients with AApoCII typically present late in life, with a median age of 70 years, with symptoms of proteinuria, which may or may not be accompanied by kidney insufficiency [61]. This late presentation is characteristic of the disease and influences management strategies. When the pathogenic variation E69V was initially identified, it brought attention to the genetic foundations of this illness [63]. The K19T mutation was later discovered to be more prevalent. Understanding familial patterns and providing genetic counseling depend heavily on these genetic discoveries. A characteristic feature of glomerular involvement in patients with AApoCII is a nodular pattern. This pattern impairs the capacity of the kidney to function normally, which might result in the known symptoms of proteinuria and perhaps escalate renal impairment [61,62].

### 3.11. Apolipoprotein C-III (AApoCIII) Amyloidosis

AApoCIII amyloidosis, one of the newest forms of the disease, was first identified in a French patient. This form of amyloidosis results from a D25V pathogenic variant in the apolipoprotein CIII gene that plays a significant role in lipid metabolism. Aside from the kidney, amyloid deposits in AApoCIII amyloidosis have been identified in the spleen, salivary glands, heart, rectum, and bronchial tubes. Patients often initially present with sicca syndrome, which precedes the development of arterial hypertension and kidney disease. The Raynaud phenomenon, pruritus, and heart involvement are additional symptoms. Patients with the D25V pathogenic variation are renowned for having hypolipidemia and, even at advanced ages, not having atherosclerotic disease. This points to a special relationship between lipid metabolism and the pathogenic variations. Patients with AApoCIII amyloidosis have had successful kidney transplants, and, remarkably, no amyloidosis recurrence has been noted in organs that have been transplanted. This could point to a potentially good prognosis for kidney recovery following transplantation [9].

### 3.12. Amyloid-ApoAIV Medullary Amyloidosis

In rare cases, ApoAIV has been identified as the principal component of amyloid deposits located solely in the kidney medulla [64], differentiating it from other forms of amyloidosis, which often involve the cortex and present with significant proteinuria. In addition to the kidney, AApoAIV amyloidosis has also been detected in the small intestine and heart and, more rarely, in the lungs and skin. AApoAIV amyloidosis may be an acquired age-related condition, similar to ATTR. It tends to misfold and accumulate in the renal medulla, likely because of the acidic and relatively hypoxic conditions of this environment [42]. The largest member of the apolipoprotein family, ApoAIV, is 46 kDa in size [102]. It is mostly produced in the small intestine and is an important antioxidant and anti-inflammatory agent in addition to its role in lipid metabolism [103,104]. Clinically, patients with AApoAIV amyloidosis experience a gradual decline in kidney function over time. This decline may accelerate in the presence of comorbid conditions such as hypertension or diabetes mellitus. A kidney biopsy followed by laser microdissection and MS is essential for diagnosis. However, the deterioration in kidney function might be mistakenly attributed to these comorbidities [42]. A kidney biopsy revealed that AApoAIV amyloidosis specifically targets the renal medulla, where substantial glassy eosinophilic Congo red-positive amyloid deposits accumulate. Often, these deposits are notably accentuated around the tubules. Unlike ALECT2 amyloidosis, the renal cortex is notably unaffected, particularly the cortical interstitium, which shows no involvement. Similarly, no amyloid deposits are observed in the glomeruli or vessels. This unique predilection for the renal medulla is also observed in AApoAI amyloidosis [105]. 

The MS signature of amyloid typically features the identification of amyloidogenic protein, SAP-component, ApoE, and, often, ApoAIV [40,106]. Since ApoAIV can also be seen in other forms of amyloidosis, IHC may not be able to correctly diagnose AApoAIV amyloidosis. One advantage of combining MS with laser microdissection is the ability to obtain a semiquantitative evaluation of the quantities of ApoAIV protein in the deposits. When AApoAIV amyloidosis is compared to other forms of amyloid where ApoAIV protein functions as a chaperone, the spectral counts of ApoAIV protein are much greater. The diagnosis of AApoAIV amyloidosis depends critically on the semiquantitation of the ApoAIV protein in amyloid plaques. Furthermore, the diagnosis of this illness entails excluding the possibility of other amyloidogenic proteins, such as AL and AHC, ATTR, LECT-2, SAA, AFib, Alys, AGel, ApoAI, and ApoAII, causing renal amyloidosis. MS is particularly effective for this purpose because it can identify and semi-quantify ApoAIV while simultaneously detecting all other primary amyloid types, thereby eliminating the need for a comprehensive and expensive IHC panel on serial tissue sections to meet this diagnostic requirement [42].

Unlike most amyloidosis types, proteinuria is typically absent or minimal (<500 mg/day). Currently, the treatment for AApoAIV amyloidosis is conservative and lacks specific targeted therapies. Accurate diagnosis is crucial to prevent the unnecessary use of chemotherapy or other targeted treatments such as stem cell transplants, which are appropriate for AL amyloidosis. Due to its location in the renal medulla and lack of glomerular involvement, AApoAIV amyloidosis does not cause proteinuria and gradually deteriorates kidney function. When early clinical indicators and particular test markers are absent, the diagnosis is frequently delayed, and, by the time kidney biopsies are performed, significant amyloid accumulation has occurred. Additionally, unlike AApoAI amyloidosis, which frequently presents with tubulointerstitial nephritis along with amyloid deposits, AApoAIV amyloidosis typically does not provoke a significant cellular response to the deposits. The diagnosis of AApoAIV amyloidosis may be overlooked if the examination is limited to the renal cortex, given that the disease affects only the renal medulla. Maintaining a high degree of suspicion and employing a Congo red stain on biopsies that include only the renal medulla can be sufficient to confirm the diagnosis of AApoAIV amyloidosis [42].

## 4. Conclusions and Future Perspectives

MS and proteome profiling have a substantial potential in the diagnosis of renal amyloidosis. By improving the amyloid typing accuracy, these techniques have made it possible to customize treatments based on the particular type of amyloid that is causing the disease. Despite these advancements, challenges remain, primarily due to the variety of amyloid proteins and the complexity of their clinical manifestations. Therapies are becoming increasingly complex due to the varying efficiency of treatments based on the kind of amyloid. Furthermore, access to sophisticated diagnostic instruments like MS is still restricted, especially in environments with minimal resources. This discrepancy indicates a critical gap in the worldwide care of renal amyloidosis and points to the urgent need for improved diagnostic methods to be more widely available. Combining larger-scale proteome investigations with NGS may help identify novel pathogenic variations and obtain a molecular understanding of the complex processes underlying amyloidosis. These discoveries may contribute to the creation of focused treatments that effectively prevent amyloidosis from progressing or even reversing its effects. Furthermore, the promise of gene-editing tools such as CRISPR presents a novel treatment option for the inherited forms of amyloidosis. These methods could change the prognosis of patients suffering from these presently incurable illnesses by directly correcting the genetic mistakes that cause amyloid formation. An international effort to standardize the diagnostic criteria and exchange resources is crucial to addressing issues related to diagnosis and treatment accessibility. Cross-border clinical trials and co-operative research could hasten the creation of treatments and diagnostic techniques that are widely available, guaranteeing fair health outcomes for all patients, regardless of where they live. As nephrology develops, the emphasis should be on improving therapeutic and diagnostic methods, as well as ensuring that these developments are available to everyone. In the global battle against renal amyloidosis, this dual strategy is essential for achieving both improved outcomes and a higher standard of living for those afflicted by this difficult illness.

## Figures and Tables

**Table 1:** Overview of diagnostic methods for renal amyloidosis.

Diagnostic Method	Description	Advantages	Limitations	Refs.
IF	Uses antibodies to detect specific proteins in kidney tissue	Direct and quick; effective for AL amyloidosis	Lower sensitivity for other types; can have false positives Need for fresh frozen tissue	[32,33,34]
IHC	Uses immunoperoxidase technique with antibodies against amyloid proteins	Good for detecting various amyloid proteins	High background staining; less effective than IF for some types	[32,35,36,37,38]
MS-based proteomics	Analyzes proteins by mass and sequence to identify and quantify amyloid deposits	Gold standard; highly accurate for all types	Requires specialized equipment and expertise	[28,39,40]
NGS	Sequences large segments of DNA or entire genomes to identify genetic variants	Provides detailed genetic insights, useful for identifying hereditary forms	Can be complex and costly; interpretation requires extensive expertise	[5,41]

Abbreviations: AL, immunoglobulin light chain; IF, immunofluorescence; IHC, immunohistochemistry; MS, mass spectrometry; NGS, next-generation sequencing.

**Table 2:** Pathogenic variants in renal amyloidosis.

Protein Type	Variant	Common in Populations	Organ Involvement	Refs.
AL	Not specified	Associated with plasma cell disorders	Various organs, including kidneys and heart	[44,45]
ATTR	V30M, V122I, T60A	Portuguese, Japanese, African Americans, Irish	Heart, nerves, occasionally kidneys	[46,47,48]
ALECT2	Not specified	Hispanic, Middle Eastern, South Asian	Kidneys, liver	[49,50,51,52]
ApoAI	Several variants	Not specified	Kidneys, heart, liver, nerves	[53,54]
AA	Not specified	Associated with inflammatory diseases	Kidneys, liver, spleen	[55]
AFib	Several variants	Not specified	Kidneys, occasionally heart	[56,57]
AGel	654G>A, 654G>T	Finnish, European, Asian, African American, Hispanic, Danish, former Czechoslovakia, France, Brazil	Eyes, skin, nerves, kidneys	[58,59]
AApoAII	Several pathogenic variants	Not specified	Kidneys, adrenals, liver, spleen, skin, rectum, bone marrow, heart	[60]
AApoCII	E69V, K19T	Not specified	Kidneys	[61,62,63]
AApoCIII	D25V	French	Kidneys, spleen, salivary glands, heart, rectum, bronchial tubes	[9]
AApoAIV	Not specified	Not specified	Kidneys (medulla), occasionally small intestine and heart	[42,64]

Abbreviations: AA, serum amyloid A; AFib, fibrinogen A α-chain; AGel, gelsolin; ALECT2, leukocyte chemotactic factor 2; AL, immunoglobulin light chain; Apo, apolipoprotein; ATTR, transthyretin.

**Table 3:** Diagnostic tools and their utility in renal amyloidosis.

Protein Type	Diagnostic Tools Used	Key Features Identified	Diagnostic Accuracy	Utility	Relative Frequency	Refs.
ATTR	MS, genetic testing	Specific mutations; fibril type	High	Identifies precise variant, critical for targeted treatment	Common in elderly, especially men	[46,47,48,66]
AA	Serum amyloid A testing, biopsy	Amyloid deposits related to inflammation	Moderate to high	Effective in linking amyloidosis to inflammatory diseases	Common in patients with chronic inflammatory diseases	[55]
AL	Immunofixation, free light chain assay, biopsy	Monoclonal light chains; amyloid type	Very high	Essential for differentiating AL from other types, guides chemotherapy	Most common form of systemic amyloidosis	[44,45]
ALECT2	Mass spectrometry, biopsy	Specific non-AL protein deposits	High	Critical for distinguishing from AL amyloidosis, often misdiagnosed	More common in certain ethnic groups (Hispanic, Middle Eastern, and South Asian)	[49,50,51,52]
ApoAI	Genetic testing, MS	Specific gene mutations; protein deposits	High	Identifies familial patterns, informs prognosis and treatment	Rare, familial	[53,54]
AGel	Genetic testing, biopsy	Mutation specific to Finnish descent, tissue deposits	High	Vital for confirming diagnosis in symptomatic patients	Rare, familial (mainly Finnish and European descent)	[58,59]
AApoCIII	MS, biopsy	D25V variant, widespread organ deposits	Moderate to high	Helps in diagnosing and understanding systemic involvement	Very rare	[9]
AFib	Genetic testing, MS	Mutation specifics, kidney-focused deposits	High	Determines risk of familial transmission and renal involvement	Rare, familial	[56,57]
AApoAII	MS, biopsy	Various pathogenic variants, widespread organ deposits	High	Helps in diagnosing and understanding systemic involvement	Very rare	[60]
AApoCII	MS, biopsy	Nodular pattern in glomeruli	Moderate to high	Aids in differentiating from other amyloidosis types	Very rare	[61,62,63]
AApoAIV	MS, biopsy	Specific medullary deposits	High	Critical for distinguishing medullary deposits	Rare, familial	[64]
ALys	MS, genetic testing	Pathogenic variants in β domain	High	Identifies specific variants, informs treatment	Rare, often age-related	[67,68,69,70]

Abbreviations: ATTR, transthyretin; AA, serum amyloid A; AL, immunoglobulin light chain; LECT2, leukocyte chemotactic factor 2; Apo, apolipoprotein; AFib, fibrinogen A α-chain; AGel, gelsolin; MS, mass spectrometry.
